# Body Fluid Collection Devices for Ostomy Patients: A Review

**DOI:** 10.3390/healthcare12212175

**Published:** 2024-10-31

**Authors:** Isaías Barbosa, Pedro Morais, Helena Torres, Jaime C. Fonseca, João L. Vilaça

**Affiliations:** 1 2Ai, School of Technology, IPCA, 4750-810 Barcelos, Portugal; pmorais@ipca.pt (P.M.); htorres@ipca.pt (H.T.); jvilaca@ipca.pt (J.L.V.); 2 Algoritmi Center, School of Engineering, University of Minho, Azurém, 4800-058 Guimarães, Portugal; jaime@dei.uminho.pt; 3 LASI, Intelligent Systems Associate Laboratory, 4800-058 Guimarães, Portugal

**Keywords:** stomas, ostomy bags, ostomy devices

## Abstract

**Background/Objectives:** Abdominal ostomy surgery has a severe impact on individuals’ daily lives. These procedures are typically indicated for conditions such as cancer, inflammatory bowel disease, or traumatic injuries. They involve creating an artificial opening, denominated the stoma, in the abdominal area to divert feces or urine, establishing a connection between the affected organs and the body’s exterior. Thus, specialized products to collect the body fluids are required, being effective and tailored products crucial to enhance the quality of life of such patients. **Methods:** This paper presents a review of fecal fluid collection devices and advanced technologies designed to assist patients with ostomies. The study aims to identify the known bags/devices and evaluate their attributed performance in enhancing the population’s physical and social quality of life. This review is based on a systematic search conducted between 20 February and 2 March 2024, in the PubMed, Scopus, Web of Science, Google Scholar, and Google Patents databases. Articles published within the last eight years from this period were included in the analysis. **Results:** The devices found in the study were classified as passive, requiring active monitoring by the user, and active, providing automated assistance. Three main categories were identified, reflecting the most significant concerns of patients: (1) devices that control fluid leakage, reducing peristomal dermatological problems; (2) devices that minimize odors and noise, reducing social embarrassment; and (3) devices that monitor fluid volume, helping with electrolyte balance, especially in patients with ileostomies. **Conclusions:** This study revealed that the existing devices meet primary collection and disposal needs. However, introducing smart devices could offer greater control and confidence to users, providing real-time information on gas pressure, stool texture, and accumulated volume. Thus, overall, the development of advanced technologies can significantly improve patients’ quality of life, restore social confidence, and enable a more effective management of the condition by sharing information with medical teams.

## 1. Introduction

An ostomy is a surgical procedure typically indicated in cases of cancer, inflammatory bowel diseases, or traumatic injuries. This procedure creates a painless opening in the abdominal region, called a stoma, allowing communication between the affected organs, such as the small intestine, large intestine, or bladder, and the exterior of the body. This opening enables the placement of a bag on the abdominal wall to collect fecal or urinary output, providing an alternative route for waste elimination [1,2,3,4]. This procedure can be temporary or permanent depending on the disease state and the patient [1,2,3,4,5]. In everyday life, patients, after surgery, when they need to relieve the contents of their intestines, cannot do so through the anus but do so through a collection bag. The collection bag is glued to the abdomen using an adhesive to protect the skin during the collection of fecal matter, with minimal discomfort for patients. These procedures include ileostomies, which involve attaching the distal segment of the small intestine (ileum) to the abdominal wall; colostomies, which involve connecting the transverse or sigmoid colon to the abdominal wall; and urostomies, which establish a connection between the urinary tract and the abdominal wall, allowing urine to bypass the urethra and drain into a stoma bag [1,2,3,4,5].

Individuals of all demographics, regardless of age or gender, may have conditions that necessitate an ostomy procedure [6]. It is estimated that in Portugal, between 10,000 and 12,000 patients are living with an ostomy [7]. Moreover, according to Hubbard et al. [4], in Europe, approximately 700,000 people live with a stoma, and more than 1 million in the United States of America (USA). The same author specifies that the figure for the United Kingdom (U.K.) is around 115,000 patients. In comparison with data from 2013, Brunicardi [8] states that in the USA, of all the neoplasms found in the American population, male patients undergoing stoma formation account for 9% of cases, while in women, the percentage is slightly lower, with 8% of all forms of neoplasms.

Living with a stoma and with a fecal fluid collection device can lead to significant embarrassment and impact various aspects of a patient’s lifestyle, often resulting in a reluctance to work, travel, or socialize with friends and family [9]. When away from home, these individuals frequently encounter challenges in locating suitable bathroom facilities for stoma care, which can exacerbate feelings of anxiety and insecurity. This constant struggle for accessibility can lead to social isolation and a diminished sense of self-worth. Thus, patients with their ostomy devices attached to the abdominal wall strive to achieve a balance between their health and emotional well-being, as they rely on the bag or device that holds their fecal fluids for elimination [10]. In this sense, the selection of a device that is both reliable and comfortable, offering effective leakage control and minimizing discomfort, is paramount to improving the patient’s quality of life.

This study aims to present the types of bags and devices currently available for patient with stomas and evaluate how effectively they improve the physical and social quality of life of the patients, as well as to present their properties. By providing a comprehensive study of fecal fluid collection devices and advanced technologies, this work aims to assess the effectiveness, usability, and impact of these devices. Moreover, by highlighting gaps in the current offerings and suggesting areas for innovation, this work aims to contribute to the state of the art in ostomy care.

## 2. Methodology

This study aims to review and summarize the types of devices available on the market for ostomy patients and to explore recent developments in the scientific literature and related projects, highlighting the characteristics that may be useful in this area for future research. For the present study, a systematic search was conducted following the guidelines of the Preferred Reporting Items for Systematic Reviews and Meta-analyses (PRISMA 2020) [11].

### 2.1. Eligibility Criteria

The eligibility criteria were assessed according to the tool’s strategy (PICO) [12], adapted to our case, in which the P for Participants corresponds to the ostomy population, the I for Intervention corresponds to the use of some model of the device, the C for Comparison corresponds to the comparison between active and passive device models in the problems of the disease, and the O for Output corresponds to the resolution of problems by the target population of the respective device they use.

In the study design, the inclusion criteria included randomized or non-randomized clinical trials and devices already implanted and on the market. Two types of devices were addressed: (1) passive devices that do not communicate with the user and (2) active devices, which communicate with the user and can be shared with medical teams in real-time. The exclusion criteria are reviews or meta-analyses, conference proceedings, or case studies.

### 2.2. Study Strategy

A search was carried out in the PubMed, Scopus, Web of Science, Google Scholar, and Google Patents libraries, 20 February and 2 March 2024, to identify relevant studies from the previous eight years. The focus was on identifying relevant studies that addressed the behavior of the ostomy population and the use of the existing products on the market, as well as the likelihood of interaction with new, more technologically advanced products. The search keywords, using the English language, in the databases consulted were “ostomy bag” OR “stoma bag” OR “colostomy bag” OR “ileostomy bag” OR “urostomy bag” OR “ostomy appliance”. The search filters included the last eight years, starting at 01012017 and ending at 31122023. In the case of Google Patents, the search dates for the same keywords were, in English, before priority: 20231231 and after priority: 20170101.

Based on these assumptions, 39 documents were found in the PubMed database, 142 in Scopus, 66 in Web Of Science, and 4200 in Google Scholar. Searching for patents using the Google Patents database, 8634 documents were found.

### 2.3. Study Selection

This search sought to find different forms of devices for ostomy patients. Duplicate records were eliminated. The titles and abstracts of the most relevant studies were selected to assess their eligibility. They were checked manually for a more detailed assessment, followed by a complete reading of the texts.

**Figure 1:**
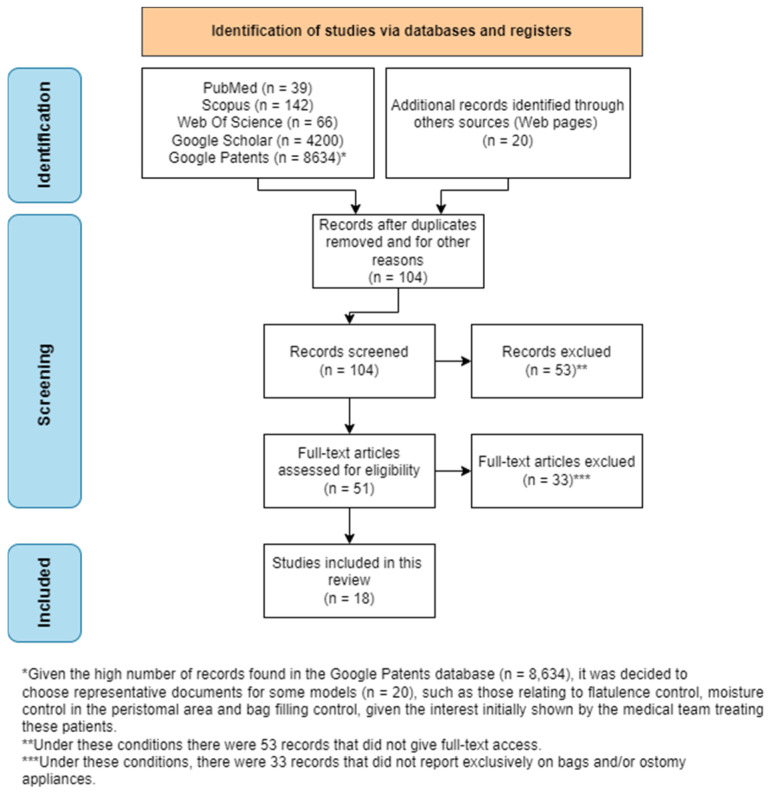
Flowchart of studies included and excluded from the Preferred Reporting Items for Systematic Review and Meta-Analysis (PRISMA) [11].

Given the high number of records found in the Google Patents database (*n* = 8634), it was decided to choose representative documents for some models (*n* = 20), such as those relating to flatulence control, moisture control in the peristomal area, and bag filling control, given the interest initially shown by the medical team treating these patients. Figure 1 shows the PRISMA-based flowchart for the eligibility of the documents worked on in this review.

After reading the abstracts, we selected documents on devices for ostomy patients, both passive and active, which we considered representative of the various techniques in use/study.

## 3. Clinical Background

### 3.1. The Stoma

**Figure 2:**
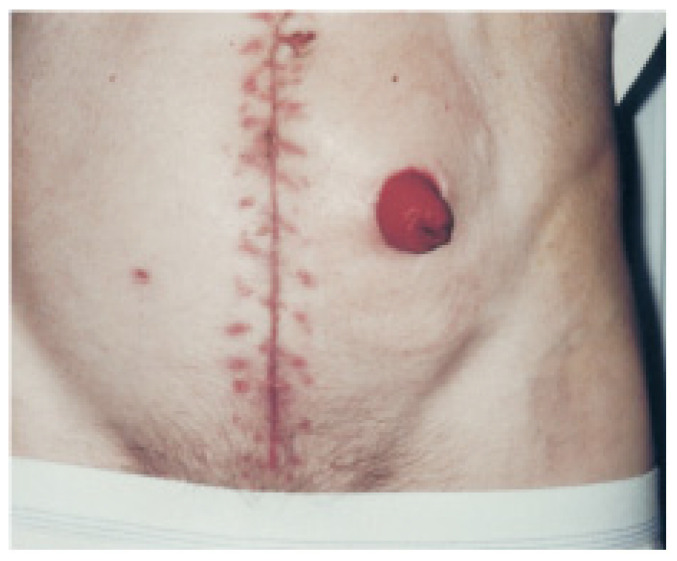
Image of a stoma, showing its red and moist appearance—image authorized by the author [15].

As previously mentioned, a stoma results from a surgical procedure that creates an opening in the abdominal region to connect the small intestine, large intestine, or bladder to the abdominal wall. This opening allows for the attachment of a pouch (bag) that adheres to the abdomen, designed to collect fecal or urinary output while protecting the surrounding skin and minimizing discomfort for the patient [6,13,14]. The typical appearance of the stoma is shown in Figure 2 [15].

### 3.2. Colostomy, Ileostomy, and Urostomy

**Figure 3:**
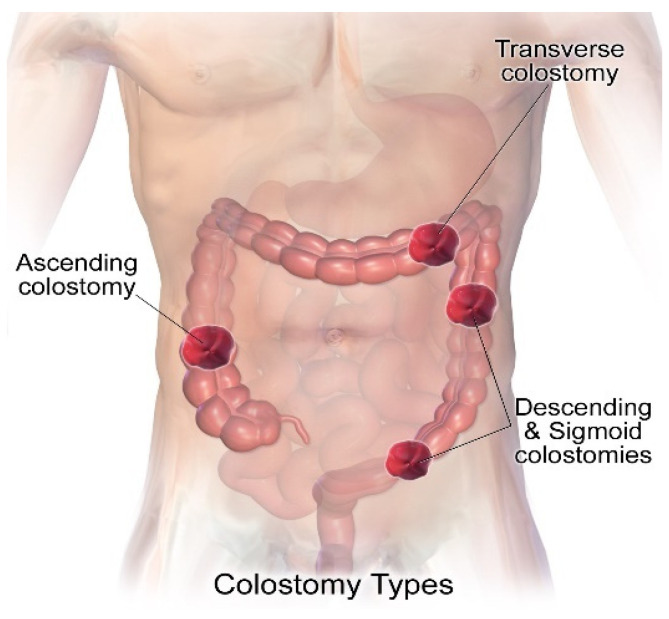
Image depicting a colostomy in the various locations where it can be implemented, depending on the type of diseased intestinal tissue. Image accessed under license from [16].

Depending on the pathology, an ostomy surgery can be called a colostomy, ileostomy, or urostomy [1,2,3,4]. A colostomy involves attaching the transverse or sigmoid colon to the abdominal wall and is typically performed as a result of localized cancer in these areas. Figure 3 shows an image of a colostomy made in the diseased part of the large intestine to be removed. The stoma is constructed in the abdominal area with a connection to a loop or to a remaining end of the intestine that does not have a disease to divert the fecal flow to the outside, which can be in the ascending, transverse, descending, and sigmoid colon [15].

**Figure 4:**
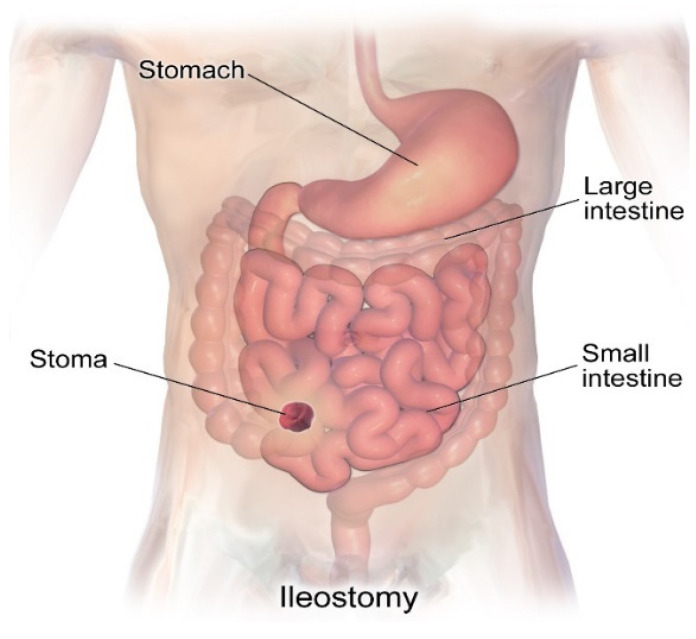
Image representing an ileostomy, in which the stoma is made in the ileum or cecum area. In this condition, the large intestine is completely discarded. Image accessed under license from [19].

An ileostomy involves attaching the distal segment of the small intestine (ileum) to the abdominal wall and is typically performed in cases of colon cancer, when the large intestine has been removed due to inflammatory bowel disease, or as a result of the malfunctioning of the large intestine, in cases of Crohn’s disease or ulcerative colitis [15]. The ileostomy, shown in Figure 4, has the same procedures as the colostomy, but now with the stoma connected to the ileum (the end of the small intestine). Throughout their lives, these patients may need to be admitted to hospital for rehydration [17,18], as the high production of fecal fluids can lead to electrolyte imbalances, which can lead to malnutrition [2].

**Figure 5:**
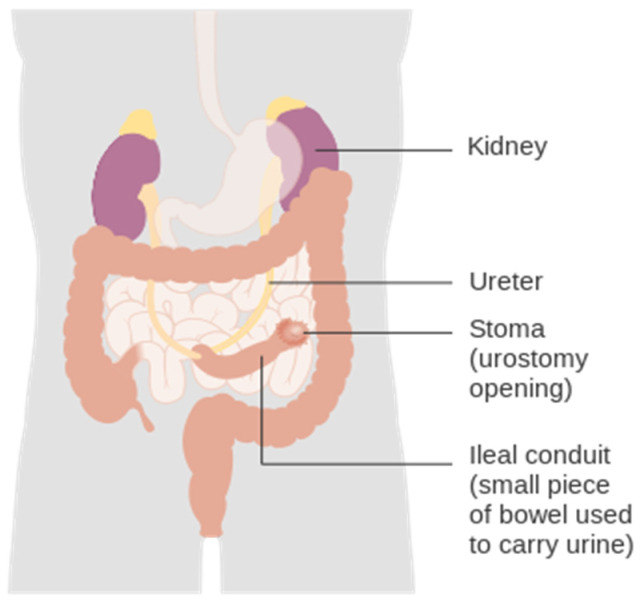
Image depicting a urostomy, in which the stoma is made with a small intestine, taken from the ileum or cecum, which connects the stoma to the kidneys, ureters, or bladder. Image accessed under license from [21].

In a urostomy, shown in Figure 5, a connection between the urinary tract and the abdominal wall into a stoma bag is performed to bypass the urethra, due to urological problems or prostate cancer. In this case, the stoma is made with a piece of small intestine, taken from the ileum or the cecum, which connects the stoma to the kidneys, ureters, or bladder, to allow urine to exit into a collection bag [6,20].

Following any of these surgical procedures, all ostomy patients require bags placed over the stoma to collect the body fluids [22]. Additionally, in cases where anastomoses are performed to reconstruct intestinal transit, stomas and bags can be temporarily used during the recovery phase when patients are unable to utilize the anal sphincter [3].

## 4. Stoma Devices

**Figure 6:**
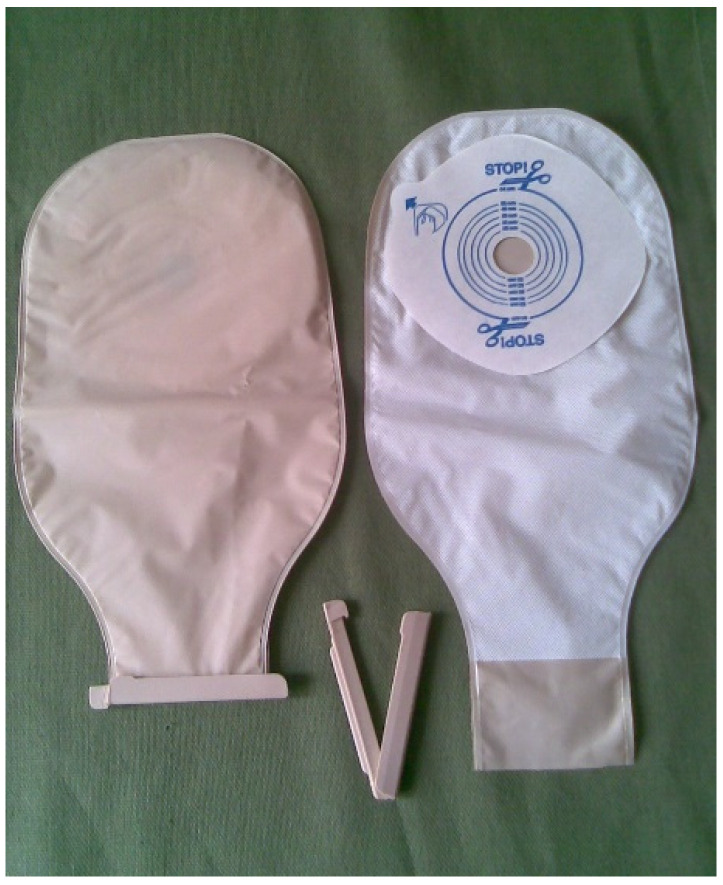
One-piece bag with purge handle. Image accessed under license from [29].

There is a wide variety of models of ostomy bags, both one-piece and two-piece. Choosing a suitable device for the stoma reduces the occurrence of complications and, therefore, reduces the possibility of losing quality of life. The choice is usually made in consultation with the medical team. If, for some reason, it loses its adherence due to poor positioning, perspiration, or obesity—allowing fluids to escape and come into contact with the skin—inflammation can occur, and this is more common in ileostomies, where the pH is more alkaline [3], with values equal to or greater than 8, while the pH value of the skin should be around pH 4.1–5.8 [23], causing local dermatitis around the stoma [24]. Figure 6 shows a one-piece bag, where the area of adherence to the skin in the peristomal area is visible, with a handle for purging (emptying the bag). This type of bag is used in ileostomies due to the large amount of fluid produced and the need to keep emptying the bag. A major embarrassment for ostomates is the fear of not knowing when the bag is complete, if it smells bad, or if the flatulence makes noise; these conditions bring them social insecurity and have a negative impact on their interactions and psychological well-being [25,26,27]. The authors Carannante et al. [28] also add the psychological burden on these patients, with the expense of the disposable tissues and bags that they have to use, causing them to waste time and money on the hygiene of their stoma.

During the research stage of this review, documents highlighting the full pouch, odor, flatulence noise, and fluid leakage were searched, as these are the situations that most distress ostomates. Afterwards, the devices identified were categorized into passive and active in relation to the patients. In the group of passive devices, there is no communication with the user. In this group, a subdivision includes one-piece bags and two-piece bags, which can also be subdivided into closed bags or bags with openings for drainage and the release of flatulence gases. The active devices allow information to be communicated with the user, and they detect various physical properties. These include the pressure of the flatulence, the volume of the material accumulated in the bag, the user’s body temperature, and the humidity of the fecal matter around the peristomal area.

Summary tables of the devices found in this review have been constructed to facilitate comparative reading between active and passive devices. Of these groups, the passive devices are summarized in Table 1, presenting the most relevant passive devices found. Active devices are summarized in Table 2, where most patents are found. These tables represent the universe of devices available for ostomy people based on the concepts and types of devices that fit into the group divisions described. In the tables, the first column lists the author describing the device or the device name. The second column identifies the target population, specifying the type of patients for whom the device is intended. The third column highlights the main features of the device, detailing its key characteristics. The fourth column outlines the device’s limitations as reported by the authors, while the fifth column explains how the device or bag was validated.

### 4.1. Passive Devices

#### 4.1.1. Ostomy Bags

**Figure 7:**
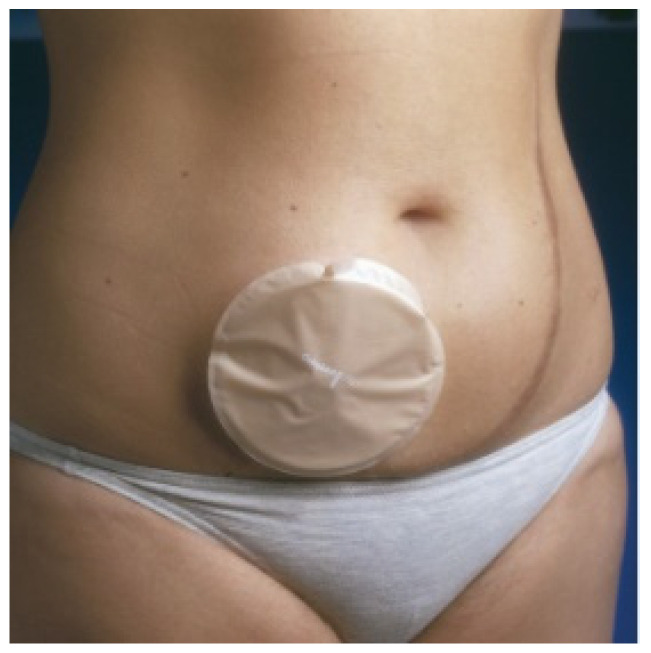
Bag for collecting fecal fluids placed over the stoma. Figure authorized by the author [14].

The image of an ostomy person is of someone using a collection bag or other means of collecting their fluids. Figure 7 shows an ostomy person with a passive collecting device on the abdominal wall, namely an ostomy bag, ready to receive fecal fluids.

**Figure 8:**
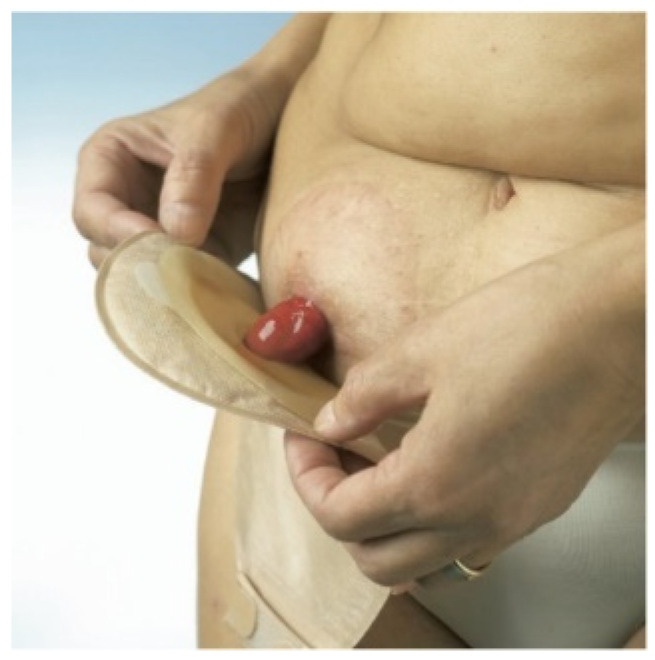
One-piece bag applied to the user’s abdomen. Figure authorized by the author [14].

The one-piece bag is normally closed and can only be used once. As it is a one-piece bag, there is less danger of the bag coming loose, as it is not reusable; it adheres well to the skin, and, consequently, the smell attributed to feces is practically unnoticeable as it is closed. It does have one disadvantage, which is flatulence, because if the bag does not have an opening to release the pressure of the gases, it ends up being uncomfortable for the user due to the volume it can occupy [2,48]. Figure 8 shows an image of a one-piece bag applied to the user’s abdomen. These bags can also be drainable when used in ileostomy/urostomy patients with a high production of more liquefied fecal products.

**Figure 9:**
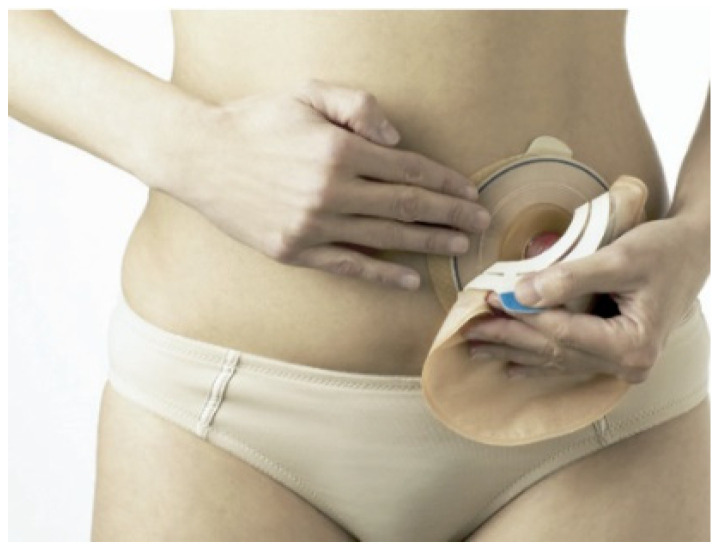
Two-piece bag, the base of which is glued to the abdomen. The bag itself can be replaced without removing the base, which is glued to the abdomen—figure authorized by the author [14].

Figure 9 shows a two-piece bag for ostomy patients to be used by a user, which can be closed or opened with drainage for ileostomies/urostomies. This is an option for the patient, with different costs to the one-piece bag.

A study by X. Li et al. [49] reveals that between 5 and 15% of fluid leaks in urostomy patients and discusses the resulting complications. In the information collected in this review, we found no references to the control of bag filling in this type of pathology, with monitoring/observation of the amount of urine collected being transferred to the patient.

With passive devices without fluid control, the user initially has no control over the fluid collection process since the stoma is open and has access to the outside, which by gravity pours the feces or urine into a bag or collection device. Over time, systems have been developed to adapt the bags to control them, as explained in the next section.

#### 4.1.2. Passive Devices with Fluid Control

**Figure 10:**
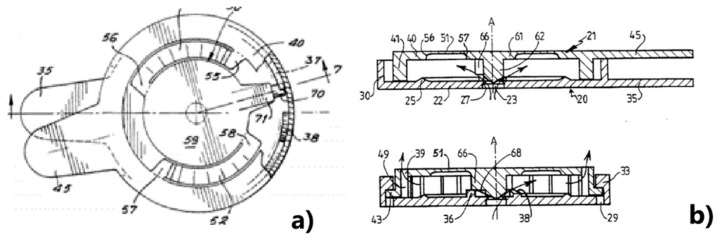
Support and cover assembly, with valve, where the letter (**a**) is the top view and the letter (**b**) is the sectional view [31].

When users want to have control over the leakage process and not be dependent on it, devices that control the fluids can be used. Figure 10 shows a device with a lid and its support to be placed in a closed bag over the stoma, in which the bag must have an opening where the valve for controlling flatulence is placed [31]. This device is intended for both colostomy and ileostomy patients. To release the flatulence, the user has to turn the lid slightly to empty the bag of the accumulated gas.

**Figure 11:**
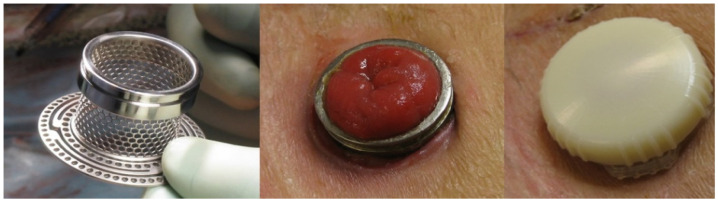
Device for ileostomy patients that seals the stoma. Figure authorized by the author [32].

**Figure 12:**
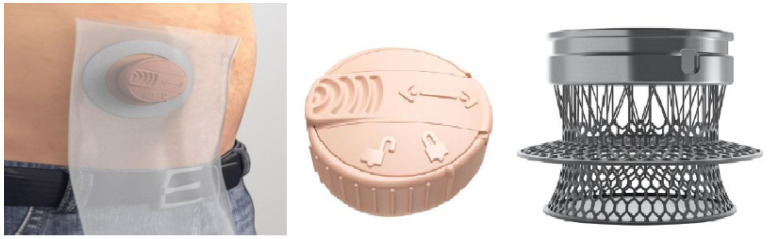
Device for ileostomy patients that closes the stoma, already approved by the CEE. Figure authorized by the author [32].

Using a different technique, a device like the one in Figure 11 was later replaced by the device in Figure 12 for ileostomies, known as the Transcutaneous Implant Evacuation System (TIES) [32], developed at the Department of Surgery at Karolinska University Hospital in Stockholm, Sweden, and currently owned by the company Ostomycure, Oslo, Norway. The titanium piece has a tubular mesh sewn between the intestine and the abdominal wall, forming a joint to allow direct access to the small intestine (ileum area). When users need to collect fluids, they pour them into a bag previously placed over the stoma. To release the flatulence, the lid is opened or loosened, allowing the gases to escape to the outside [33]. The devices in Figure 11 and Figure 12 are invasive (embedded in the abdominal wall), and the target audience is ileostomy patients.

**Figure 13:**
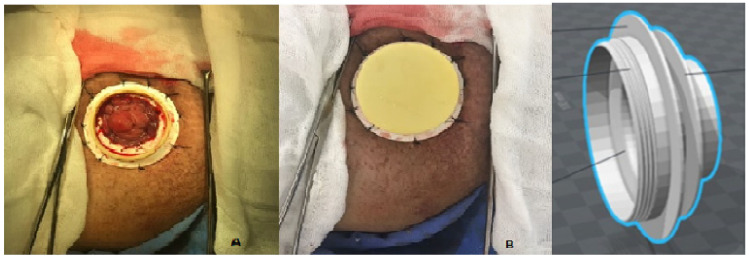
Device for ileostomy patients called VITAE [34]. Image accessed under license from http://creativecommons.org/licenses/by/4.0.

The Valve Intestinal Type Artificial Sphincter (VITAE) project, shown in Figure 13, also follows the same principle as the TIES project. This project is being developed in Mexico, in the Oncological Surgery Department of the Juarez Hospital in Mexico City, by the authors Álvarez et al. [34]. It is also an invasive application and can be closed by a cap for ileostomy patients. We note that this device is still in the development phase.

Another solution that has been found but is still in the design phase comes from Australia and is called StomaLife. It resembles a faucet, which, when opened, is used to release and eliminate waste. It consists of two parts: one part has a crown of magnets, which is placed inside the ostomy person’s abdominal wall through a surgical operation, and the second part, which is mobile, is placed over the stoma with a tap. As it has a magnet, the assembly forms a single piece by magnetic attraction, i.e., the external part will be connected to the internal part without adhesives [35]. According to the author, the user decides when and how best to dispose of their fecal products, giving them “greater security” in the hygiene process associated with the stoma. This solution closes the stoma, avoiding odor, noise, and leakage problems. This project is aimed at ileostomy patients. The state of development of this device is currently unknown.

The devices found with the possibility of control, which insert a foreign body into the patient’s abdominal wall, can induce the likelihood of inflammatory situations and possible rejections/complications. In the case of inflammation, this is treated with antifungal and or inflammatory creams. Burch [50] states that it is impossible to anticipate a complication, such as an allergy to a device inserted into the human body, because it can happen in many ways. In the case of rejection, with the VITAE project, there were problems in the absorption/acceptance of the titanium parts that make up the devices, since at the junction of the abdominal wall with the intestine and the connection to the device, it ended up having flaws and not having a perfect union, giving rise to leaks.

**Figure 14:**
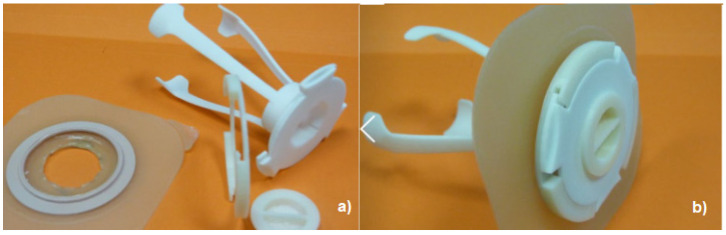
Project by the University of Oviedo, with a new approach to the relationship with the stoma. Figure authorized by the author [36]. (**a**) Represents the parts that make up the project: the adhesive fastener, the bag holder and the lid. (**b**) Assembled without the bag.

In a different way of approaching the stoma, the study by Sierra et al. [36] from the University of Oviedo, Spain, presents a prototype shown in Figure 14a,b. Figure 14a shows four parts: the skin attachment ring + adapter, the applicator (with three legs), and the closure cap. Figure 14b shows the device with assembled parts from Figure 14a.

**Figure 15:**
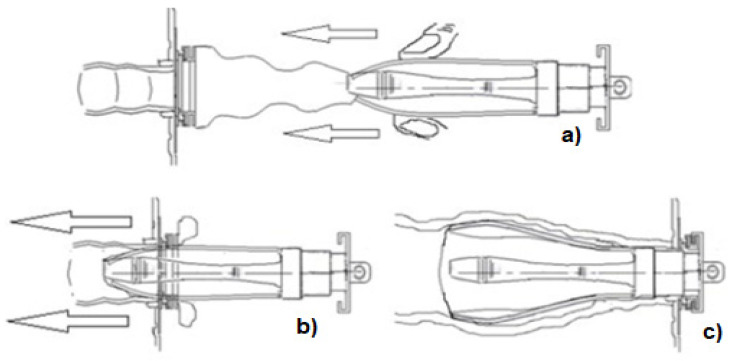
Passive device for ostomates, with no leaks or odors. It works inside the user’s intestine—figure authorized by the author [36]. The letters (**a**–**c**) show the dynamics of mounting the device on the stoma. In (**a**) the assembly before being inserted into the stoma, where the fingers are an important part of adjusting the device to the stoma. In (**b**) the beginning of the insertion of the device into the stoma and in (**c**) the assembled device, already inside the stoma.

Figure 15 shows the working method of Sierra et al. [36] with the letter (a) being the part corresponding to the stoma, where the fixing ring is applied and where a bag is assembled and fixed. The bottom of the bag is also attached, by finger pressure, to the applicator to be pushed by the user into the intestine through the fixing ring glued to the stoma, which corresponds to the letter (b), with the bag and applicator remaining inside the intestine. The stoma is closed with the cap, as shown in letter (c). According to the authors, there is no leakage or odor with this procedure, as the system remains closed. When it is necessary to remove the fecal fluids, the procedure is reversed, starting by unlocking the applicator from the fixing ring and removing the applicator with the fluids inside the bag. When the bag is outside the intestine/stoma, the dirty part has no contact with the user, as it remains inside the bag. According to the authors, it can be used in colostomies or ileostomies. It is considered an invasive device.

**Figure 16:**
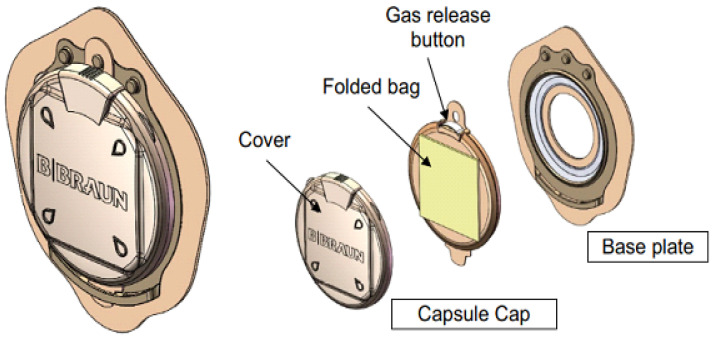
Low-profile two-piece stoma control device [37]. Image accessed under license from http://creativecommons.org/licenses/by/4.0/, accessed on 1 January 2024.

**Figure 17:**
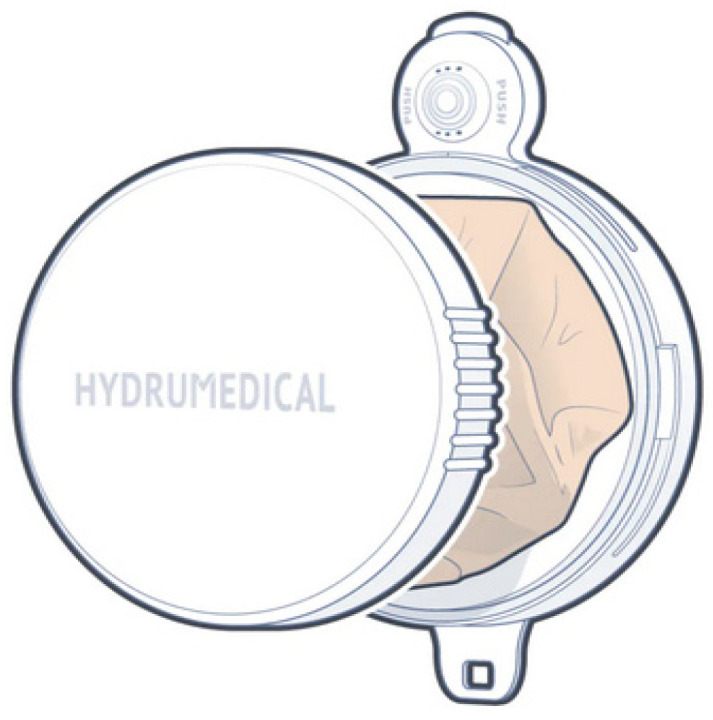
Hydrustoma© C3. Figure authorized by the author [38].

A different concept of stoma control, without invasive methods, can be seen in Figure 16 and Figure 17. In the first case, the project originated at the University Hospital Center of Nantes, France, with a two-part device. The base is glued to the body with an adhesive, and the capsule containing a folded bag is only visible if the lid is lifted, through which the fecal fluids exit. The profile of the device is low so that it can be hidden under clothing. For the release of gases (flatulence), the device has a button (valve) which, when pressed, releases the pressure. According to the authors, the project was well accepted by the target population in the experimental phase and was used in patients with colostomies (ileostomy/colostomy) [37].

In the second case, Figure 17, Hydrumedical, a company that produces medical articles in Portugal, presents a solution for ostomates under the name Hydrustoma© C3 [38]. The page supporting the project assumes that the user controls their colostomy. The operating principle is similar to Figure 16. It also has a button that allows flatulence gases to escape when pressed.

In this device, the fecal product collection bag is also folded and stored under the lid. The difference lies in the opening of the lid: in the University of Nantes project, the lid is raised, and in the Hydrumedical project, the lid is threaded with a ¼ turn. The company’s website indicates that it will be available soon.

**Figure 18:**
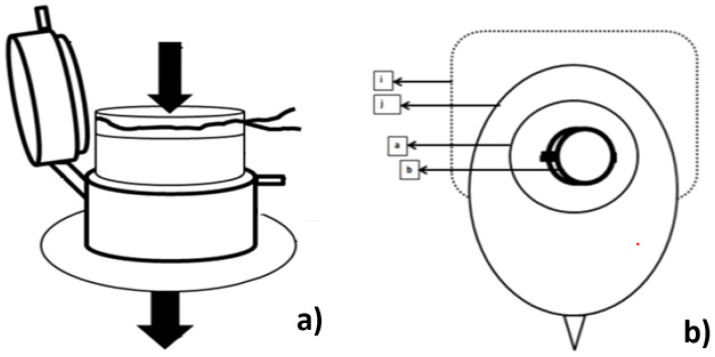
Device that can be attached to a stoma bag using the fixing plate. Image (**a**) shows the device complete with cover. Image (**b**) shows the device mounted on the attachment plate of a two-piece bag. The letters in this image identify (b) the top of the device without the cover, (a) the body of the device, (j) the tab to be placed on the fixing plate, and (i) the fixing plate of an ostomy bag already available on the market [39]. Image obtained under license from http://creativecommons.org/licenses/by/4.0/, accessed on 25 February 2024.

The device in Figure 18 is a different application used to help with postoperative ostomies. According to the authors Guzelyuz B. and Uludag S. [39], this device facilitates the hygienization of the stoma. It prevents leakage onto the skin and reduces inflammation in the peristomal area, which contributes to a faster recovery for the patient. This is due to the device’s ability to be adjusted to the stoma with a minimal chance of leakage onto the skin. It uses ostomy bags already on the market, taking advantage of the plate glued to the skin in the peristomal area.

Figure 18a shows the complete device, which includes a flap at the back and an outlet at the top, which can be closed with a lid. Figure 18b shows the device already mounted on the plate, which is positioned over the stoma and fixed to the abdominal area using an adhesive.

### 4.2. Active Devices with Fluid Control

Considering active devices, those with a technology that establishes some form of communication with the user, they are distinguished by being able to include multiple sensors, as they can detect more than one property relating to the control/information process, with cumulative characteristics of pressure/flatulence control, the calculation of fecal matter in the bag, and temperature, among others. Within this group of devices, the subdivisions presented in this research are skin leakage control, flatulence control, and bag filling control, with descriptions presented below.

#### 4.2.1. Monitoring/Control Devices for Skin Spills

Starting with active devices that monitor/control the leakage of fluids onto the skin around the stoma, in this group, we find the inventors Hansen et al. [40], with a patent registered since 2019. It is a device to be placed over the stoma that only detects if fluid has leaked onto the skin in three concentric zones in the peristomal area. The device has been updated over time with the application of new patents. The document consulted presents drawings of sensors to be implemented in the dressings and only explains how to detect possible fluid invasion in the areas under control. It requires a collection bag and does not specify whether it is for colostomies or ileostomies.

The inventors Seres et al. [41] have filed a patent application under the name Ostomy Application System, which is a system that monitors possible flooding in the peristomal area. According to the authors, it can cumulatively measure more physical properties, such as body temperature and fecal matter inside the collection bag. It does not specify whether it is intended for colostomy or ileostomy patients.

Another device called the Ostomy Device with Leak Detection, invented by Michel P. et al. [42], also aims to solve the problem of detecting flooding in the area around the stoma due to pH differences and the consequent inflammation/irritation of the skin. In this case, it uses soluble conductive ink that dissolves when exposed to moisture. It uses radio frequency identification (RFID) technology to communicate with the processing part of the device. Whether the technology applies to devices for colostomy or ileostomy patients is not specified.

The Danish author Knoedler [43] has filed a worldwide patent for a device with sensors, which also includes the detection of fluid leaks into the peristomal area and or excessive perspiration on the user’s part. Under these conditions, the user is alerted to the possibility of a break in the fixation of the collection bag in the abdominal area. The same device checks whether the stoma has leaked into the peristomal area, thus preventing possible inflammation of the user’s skin. It does not specify whether it is intended for colostomy or ileostomy patients.

**Figure 19:**
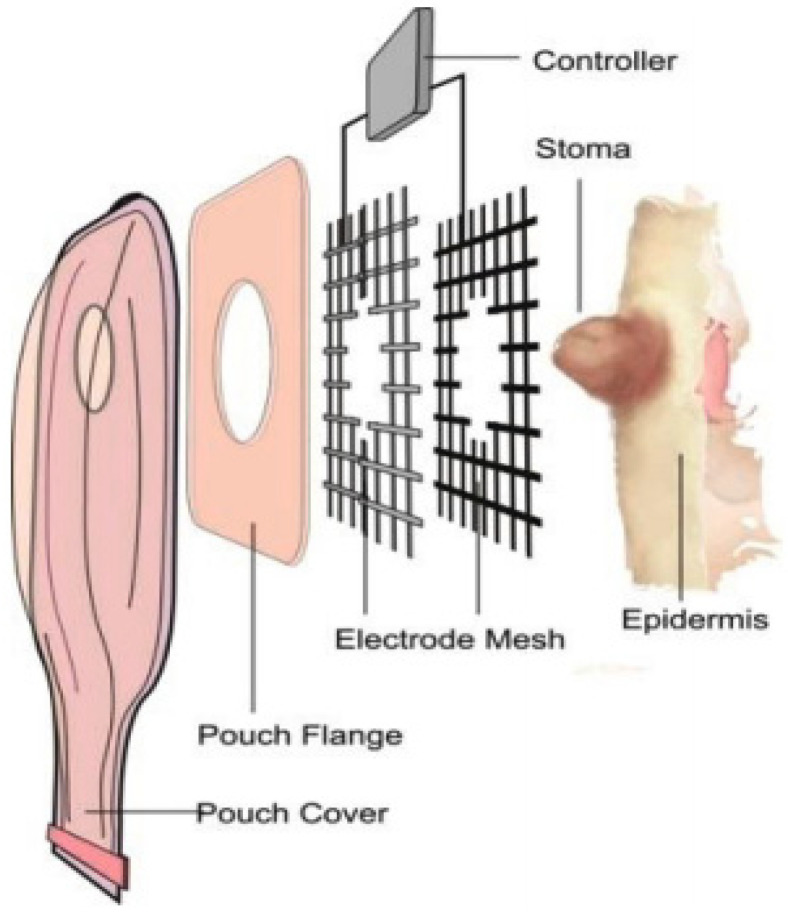
pH control, using a “hydrocolloid tablet” impregnated with polytryptophan, activated by a square wave current [51]. Figure authorized by the editor.

Another project, now by McLister and Davis [51], from the University of Ulster, Northern Ireland, shown in Figure 19, presents a treatment for skin pH in areas invaded by fluids in the peristomal area, using a grid-shaped “hydrocolloid tablet” impregnated with polytryptophan. According to the authors, the process is activated by a current with a square waveform and an intensity of a few nanoamperes (nA). The article does not specify the frequency, time, working voltage, or energy consumption used to balance the pH. It is not specified whether the device is intended for colostomy or ileostomy patients.

#### 4.2.2. Flatulence Monitoring/Control Devices

In the group of flatulence monitoring/control devices, the patents of Seres et al. [41] and Knoedler [43] were used to control leakage zones around the stoma and the control of flatulence. We can add to this group the inventors Kralovec et al. [44], with the device they patented under the name Ostomy Silencer and Method, which has the particularity of eliminating the sound produced by flatulence. When reading the patent, the author only describes the concept, stating that the “sound-canceling electronics” may include a “threshold detector”. He does not explain much about the process but mentions that it is a device for colostomy patients.

#### 4.2.3. Bag Filling Monitoring/Control Devices

In the area of monitoring/controlling the filling of ostomy bags, the target group for this technology is patients with ileostomies, who need to control the amount of fluid deposited in the bag (between 600 and 800 mL/day [52] or even 2000 mL/day [53]) to achieve electrolyte rebalancing with the fluid intake required by their bodies.

**Figure 20:**
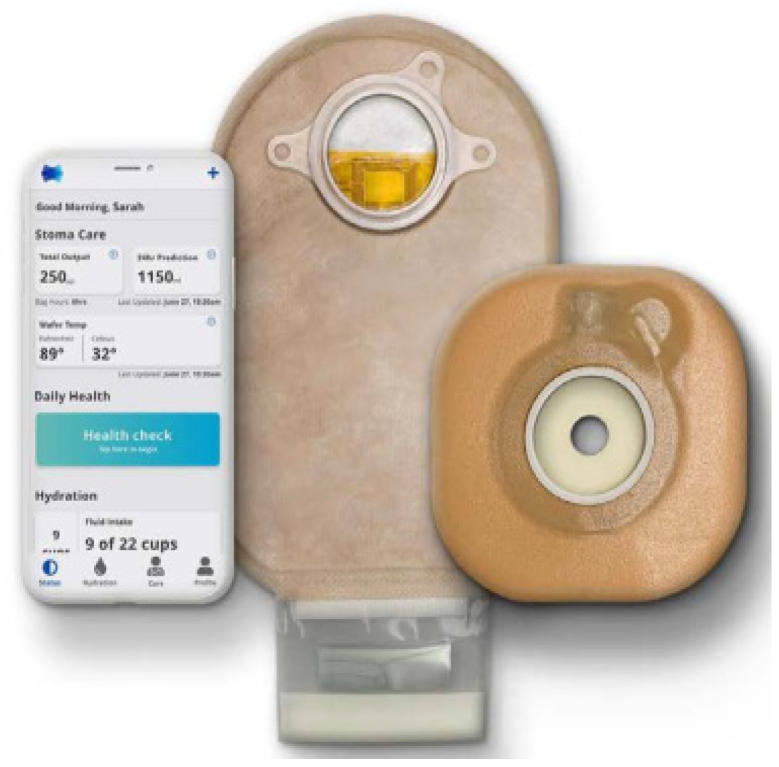
Alfred Smart Bag device, where the mobile application and the two-piece bag are visible, with the implementation of resistive/capacitive sensor arrays, calculating the amount of waste in the bag. Figure authorized by the editor [55].

The Alfred Smart Bag device in Figure 20, by the authors M. Seres and A. Bloom [54], claims to be the world’s first intelligent ostomy bag, with the ability to calculate the amount of fecal product in the bag and monitor the condition of the stoma [41], preventing the patient from becoming dehydrated. Figure 20 shows the device, consisting of a collection bag, where resistive/capacitive sensor arrays are mounted to detect the amount of fluid in the bag, and an image of the accompanying mobile application. The processing of the sensory data and the communication device are mounted on the collection bag. According to the authors, this device is intended for ileostomy patients.

**Figure 21:**
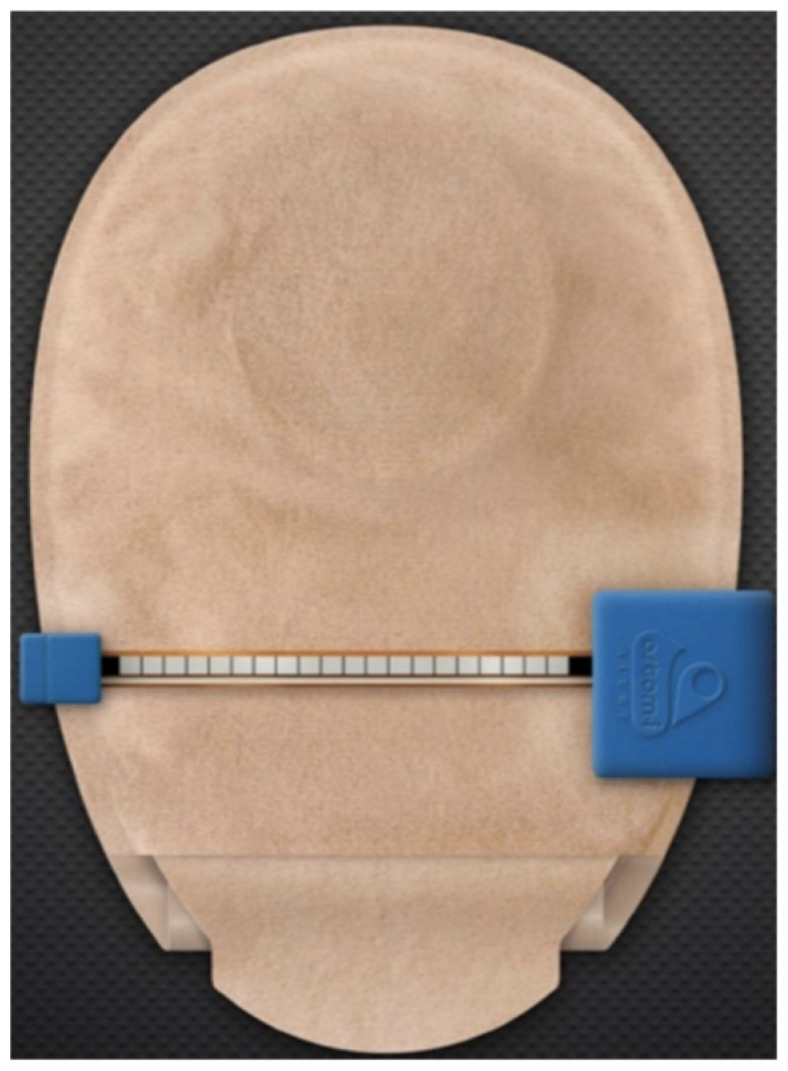
Bag with a device that measures the volume of the fecal product [44]. Image accessed under license from http://creativecommons.org/licenses/by/4.0/, accessed on 25 February 2024.

In a study by Kontovounisios et al. [53] with these patients, the device shown in Figure 21 was used, known as Ostom-i (the device at the time of the investigation belonged to the company “11 Health”), which uses a flexible piezoelectric sensor that provides the user with the amount of fluid in the bag by deforming the bag through the action of the deposited material. With this type of device, the user is informed with an error of 10% via Bluetooth of the amount of fecal product to be eliminated and can thus decide what to do depending on the information received about their electrolyte rebalancing.

**Figure 22:**
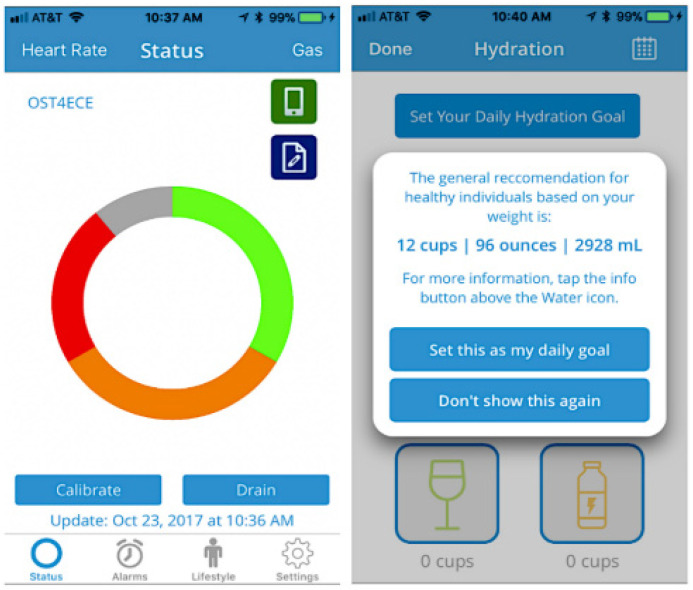
Mobile application of the Ostom-i biosensor [47]. Image accessed under license https://creativecommons.org/licenses/by/4.0/, accessed on 25 February 2024.

Figure 22 shows the mobile application accompanying the device, where monitoring the individual’s hydration is possible.

**Figure 23:**
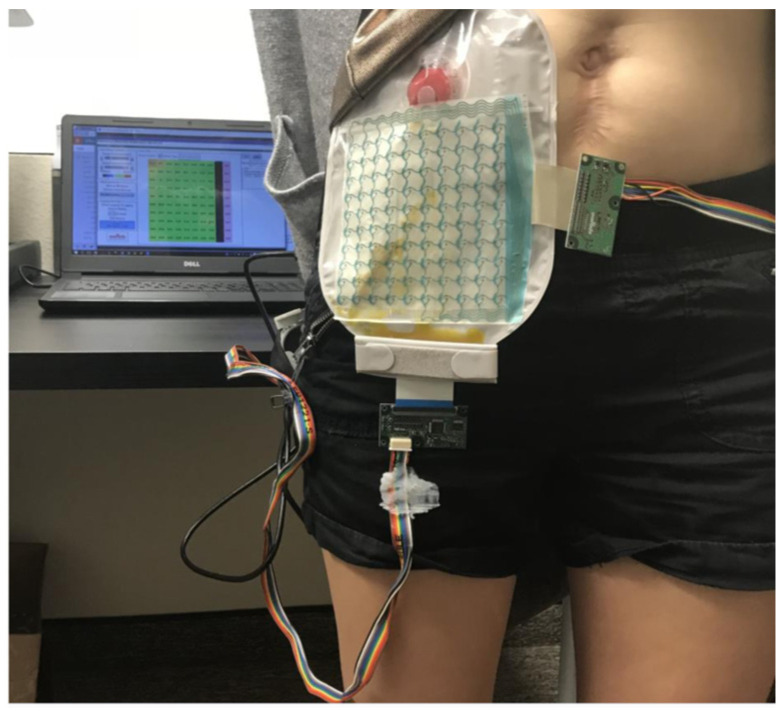
Test phase of a bag that measures the amount of fecal fluid [45]. Image accessed under license https://creativecommons.org/licenses/by/4.0/, accessed on 20 February 2024 (CC BY 4.0).

Measuring the amount of fecal product deposited in the bags, Fearn et al. [45] present a technology using a matrix network of Negative Temperature Coefficient Thermistors (NTCs) placed on a bag, as shown in Figure 23. This figure shows a prototype for measuring the amount of fluid and thus being able to determine, by the heat of the fluids, which area of the bag is being invaded by calculating the volume.

## 5. Discussion

With the documentation collected, it was possible to review various devices for ostomy patients, addressing issues such as controlling the noise and odor of flatus, managing fecal fluids, and protecting the peristomal area.

The variety of bags and devices identified in this research offers solutions that meet the needs of ostomy patients. According to the initial classification between passive and active devices, the former allows the user control over the stoma, enabling the release of fluids and gases as required. On the other hand, in options without this control, the user must constantly monitor the state of their fluid collection bag. Active devices, on the other hand, which are more technologically sophisticated, feature sensors whose physical properties are designed to mitigate common problems in this population, such as the invasion of the peristomal zone, the noise and odor of flatus, and the management of the volume of fluids in the bag.

The damage caused to the skin by the loss of fecal fluids in the areas around the stoma is characterized by inflammation of the epidermis, with intense and persistent symptoms of pain, itching, and a burning sensation [56]. These problems occur when, for some reason, the fluids leak through to the skin. The devices evaluated in this study, which have leakage control, such as TIES, VITAE, and StomaLife, have proven effective in preventing these incidents, offering significant support to patients. In the case of the VITAE project [34], the device had to be removed from the volunteer’s body due to difficulties adhering to the abdominal area, leading the project to a re-analysis phase. On the other hand, the StomaLife project [35] takes a different approach to solving the problem. However, it is currently not visible online, with only a video explaining the concept and no clear information on the state of development or whether it has faced problems in implementation or has gone no further than a preliminary intention. Another innovative initiative is that of Sierra et al. [36], which involves introducing the bag directly into the intestine via the stoma, a technique that, if successful, could solve the problems of flooding the peristomal area and odor. For this project, it is known that tests have begun, although the results are not yet known. The project by Guzelyuz B. and Uludag S. [39] also contributes significantly to the rapid recovery of patients in the postoperative period by providing the effective control of leakage in the peristomal zone, according to the authors.

According to Duluklu et al. [57], the odor of fluids and flatus is attenuated in passive devices through filters or fragrances such as oils or lavender, which help to minimize the odor when it is released randomly without control by the patient. Controlling the noise produced by flatulence and the odor is only effectively accomplished using passive devices with tools, as in Lehur et al. [37] and the Hydrustoma^®^ C3 [38].

Devices with sensors allow users to control their stomas more effectively. Fearn et al. [55] point out that more than 55% of ileostomy patients face complications from dehydration. In a study by Rouholiman et al. [47], a significant reduction in hospital readmission rates was observed, from 14.9% to 2.4%, thanks to the use of the Ostom-i device, which contributes to the rebalancing of electrolytes and the better control of hydration. Kontovounisios et al. [53] corroborate the reduction in hospital readmission rates using the same device. The Alfred Smart Bag device, developed by Seres et al. [54] is also part of this group and stands out for its control of rehydration.

Although the patents evaluated in this investigation offer solutions that meet the needs discussed, the lack of confirmatory data, the general omission of validations, and their limitations are still outstanding issues.

## 6. Conclusions

Products for ostomy patients are being improved. Active products are the most advanced, the result of current technology with more personalized sensors, and the closest to the needs of these patients. Research centers, inventors, researchers, and the industry have been attentive to these needs, with increasingly sophisticated solutions. In active devices, real-time communication with the patient or the medical team about what is happening at the mouth of the stoma, with the medical parameters observed, can prevent problems without needing to go to the hospital. From the patient’s side, this would mean less financial and social costs and a better health and quality of life, regarding their self-confidence and social acceptance.

## 7. Future Work

We believe that there is room for new technological solutions, with sensors designed for this purpose that respond to the difficulties encountered, such as controlling leakage into the peristomal area, the noise of the flatus, and the amount of liquid deposited in the bag. These conditions, in part or in whole, if left unchecked, are responsible for the social distancing of part of this population.

## Figures and Tables

**Table 1:** Overview of passive devices for ostomy patients.

Author(s) of the Invention (Year)	Target Public (Patients)	Main Characteristics	Limitations	Validation
Coloplast [30] (2021)	Colostomy, ileostomy, or urostomy patients	Fecal fluid collection bags	They do not allow control of fecal fluid loss	It is unknown, but it is a commercial product.
H. Holtermann et al. * [31] (1993)	Colostomy or ileostomy patients	The device is to be applied to the closed pouch of an ostomy person to control flatulence.	A collection bag is required	Unknown.
K. Strigård et al. [32] (2011)	Ileostomy patients	The Transcutaneous Implant Evacuation System (TIES) is a titanium device. It attempts to fuse the intestine to the abdominal wall. The user controls the outflow of feces by removing a cap so that the fecal content leaks into a bag.	This device is subject to rejection by the human body, as it is invasive. It is inserted by surgery	A few volunteers were chosen from a group of ileostomy patients, and the device was applied for testing. The tests are still being carried out.
Ostomycure [33] (2023)	Ileostomy patients	A new model of TIES (Transcutaneous Implant Evacuation System). The user controls the exit of feces by removing a lid so that the feces are discharged into a bag.	The problem of rejection of the titanium piece by the patient’s body seems to have been solved.	Once the testing phase ended, it was advertised as a commercial product on the website: https://ostomycure.com, accessed on 21 December 2023.
C. E. Álvarez-Ponce et al. [34] (2020)	Ileostomy patients	Project name VITAE (artificial sphincter-type intestinal valve). The user controls the elimination of feces by removing a lid to empty the feces into a bag. This is a similar project to TIES.	The difficulties of total adherence of the intestinal mucosa to the device have been a bias. According to the authors, more studies are needed	It was implanted in a patient and later removed at the patient’s request.
S. Sabeti [35] (although this work it is outside the search window, it shows another technique used. The project was already known in 2012, but has since ceased to be traceable on the internet, with a demonstration film at https://gust.com/companies/stomalife, accessed on 14 December 2023) (2012)	Colostomy or ileostomy patients	The name of the project is StomaLife. The device is inserted into the stoma and is held in place by a magnet implanted during the surgery, under the abdominal wall, magnetically attracting the outer part that has the fecal control tap. No adhesive is used to keep the device attached to the abdominal area.	They are unknown	They are unknown. The project still seems to be in the development phase.
J. M. Sierra et al. [36] (2020)	Colostomy or ileostomy patients	The device and its bag are inserted into the intestine through the stoma. When the patient decides to eliminate the feces from the intestine, they remove the device that carries the feces to be eliminated in the bag.	They are unknown	Tests with users have already begun, but the results are still unknown.
P. A. Lehur et al. [37] (2019)	Colostomy patients	According to the authors, it is an alternative to using bags and can improve intestinal control and acceptance of the stoma. It also controls flatulence. A better quality of life has been reported with this device.	They are unknown	Several users have tried out the device. The authors report that it has been very well received and call for more tests to be carried out.
Hydrumedical, [38] (2021)	Colostomy or ileostomy patients	According to the website behind it, this is an innovative solution in which the colostomy patients take complete control of their colostomy. They also have control over their flatulence.	They are unknown	They are unknown.
B. Guzelyuz et al. [39] (2023)	Colostomy or ileostomy patients	According to the document evaluated, this device will be used where protection is needed for the stoma area, peristomal leakage, and subsequent skin irritation.	They are unknown	They are unknown.
* patent				

**Table 2:** Overview of active devices for ostomy patients.

Author(s) of the Invention (URLs) (Year)	Target Public (Patients)	Main Characteristics	Limitations	Validation
J. A. Hansen et al. [40] (2019) *	The packaging states that it can be used on stomas.	The device controls three concentric zones in the leakage of fluids to the skin through the stoma, sending alarms to the system via communications (it does not specify the type). It talks about memories, processors, a first interface, and a second interface, among other things, without specifying.	They are unknown	They are unknown
M. Seres et al., [41] (according to the authors, the device is already on sale to the public) (2019) *	It does not specify whether it is intended for ileostomy or colostomy patients.	A multisensory device also controls the leakage zones near the stoma by concentric zones.	They are unknown	They are unknown
P. N. Michael et al. [42] (2020) *	It does not specify whether it is intended for ileostomy or colostomy patients.	It detects flooding in the stoma area, using soluble conductive ink that dissolves when exposed to moisture in the areas concentric to the stoma, sending closed/open information to the transponder antenna. It uses radio frequency identification (RFID) technology to communicate between the detection and processing zones. The circuit is closed when there is no flooding and opens when the ink dissolves in that or other areas.	They are unknown	They are unknown
S. Knoedler [43] (2020)*	It does not specify whether it is intended for ileostomy patients or colostomy patients.	A multisensory device that also monitors leakage zones near the stoma by concentric zones detects moisture near the adhesive that supports the device in the stoma area, anticipating possible failure of the adhesive plate and the consequent collapse of the device.	They are unknown	They are unknown
M. E. Kralovec et al. [44] (although this work is outside the search window, it shows yet another technique used) (2015) *	Colostomy patients.	The patent states that it has the particularity of eliminating the sound produced by flatulence. It is referred to as a device for colostomy patients and consists of a loudspeaker, a microphone, and the electronics of the cancellation circuit.	They are unknown	They are unknown
R. Fearn et al. [45] (2020)	Ileostomy patients.	The user can use a flexible piezoelectric sensor to determine how much liquid is in the bag. With this device, the user is aware, via Bluetooth Low Energy, of the amount of fecal product to be eliminated, so they can decide what to do with the information received, whether they need to dehydrate or not.	This device was considered acceptable and usable but did not provide an accurate and consistent reading due to connectivity problems and a high noise/signal ratio in a dynamic environment	Ileostomy volunteers
M. Seres et al. [46] (2018)	Ileostomy patients.	A device in which sets of resistive/capacitive sensors are mounted, capable of calculating the number of stools in the bag, with thermal sensors indicating the risk of dehydration. It can also record skin conditions such as irritation.	It is not known	Several studies have been carried out with ileostomy volunteers
D. Rouholiman et al. [47] (2018)	Colostomy patients, Ileostomy patients, Urostomy patients.	A portable biosensor has been built and connected via Bluetooth, making measuring the flow rate/amount of fluid in the ostomy pouch easier.	According to the authors, the Ostom-i alert sensor can improve users’ quality of life by giving them the freedom and confidence to participate in daily activities, knowing that they can check if their ostomy bag is full discreetly and privately	Twenty volunteers with ostomies were recruited, and the test was run for one month. Volunteers who had had ostomies for less than six months before the test date were excluded
* patent				

## Data Availability

Data are contained within the article.
